# Changes in the endemic-epidemic pattern of malaria in Colombia, 1978-2021

**DOI:** 10.1590/0037-8682-0364-2023

**Published:** 2024-04-22

**Authors:** Julio Cesar Padilla Rodríguez, Mario Javier Olivera, Luis Acuña Cantillo, Pablo Chaparro-Narváez

**Affiliations:** 1 Red de Gestión de Conocimiento, Investigación e Innovación en Malaria, Bogotá D.C., Colombia.; 2 Instituto Nacional de Salud, Grupo de Parasitología, Bogotá D.C., Colombia.; 3 Instituto Nacional de Salud, Grupo de Entomología, Bogotá D.C., Colombia.; 4 Instituto Nacional de Salud, Observatorio Nacional de Salud, Bogotá D.C., Colombia.

**Keywords:** Endemic, Epidemic, Malaria. Mortality, Plasmodium vivax, Plasmodium falciparum

## Abstract

**Background:**

Malaria is a major global public health issue with varying epidemiologies across countries. In Colombia, it is a priority endemic-epidemic event included in the national public health policy. However, evidence demonstrating nationwide variations in the disease behavior is limited. This study aimed to analyze changes in the levels and distribution of endemic-epidemic malaria transmission in the eco-epidemiological regions of Colombia from 1978 to 1999 and 2000 to 2021.

**Methods:**

We conducted a comprehensive time-series study using official secondary data on malaria-associated morbidity and mortality in Colombia from 1978 to 2021. Temporal-spatial and population variables were analyzed, and the absolute and relative frequency measures of general and regional morbidity and mortality were estimated.

**Results:**

We observed an 18% reduction in malaria endemic cases between the two study periods. The frequency and severity of the epidemic transmission of malaria varied less and were comparable across both periods. A shift was observed in the frequency of parasitic infections, with a tendency to match and increase infections by *Plasmodium falciparum*. The risk of malaria transmission varied significantly among the eco-epidemiological regions during both study periods. This study demonstrated a sustained decrease of 78% in malarial mortality.

**Conclusions:**

Although the endemic components of malaria decreased slightly between the two study periods, the epidemic pattern persisted. There were significant variations in the risk of transmission across the different eco-epidemiological regions. These findings underscore the importance of targeted public health interventions in reducing malarial morbidity and mortality rates in Colombia.

## INTRODUCTION

Malaria is a persistent public health concern worldwide. According to the 2022 World Malaria Report, 247 million cases of malaria were estimated across 84 endemic countries in 2021, an increase from 245 million in 2020. The Americas contributed to only 0.2% of the total global malaria cases in 2021, when the total number of malaria cases reduced by 60%^1^. 

In Colombia, malaria is transmitted in an endemic-epidemic manner, resulting in an unstable transmission pattern. Its endemic-epidemicity increase was largest during the late twentieth century and early this millennium, although a reduction close to 40% was observed in the accumulated case-load during recent decades^2^.

Malaria transmission in the Americas is characterized by persistent endemic-epidemic patterns, heterogeneity, and unstable transmission of varying intensities. This complex event occurs in areas where populations, parasite carriers, and vectors interact and are influenced by demographic and economic processes as well as political and social conflicts. Throughout the mid-twentieth century, various initiatives and projects were implemented in the region to control and eradicate malaria, producing successful short-term results. However, these efforts were not sustainable^3^ ^,^ ^4^. 

Despite the experience gained from managing territorial malaria programs and evidence resulting from recent prevention and control projects and applied research conducted by national experts, research centers, and universities, these experiences could not be scaled well, and they were hardly considered as local evidence for the definition of objectives and goals in policies, plans, and programs for malaria elimination in the country^5^ ^,^ ^6^. 

This study aimed to describe the changes observed in the levels and distribution of endemo-epidemic malaria transmission in the eco-epidemiological regions of the national territory and to estimate the possible determinants of these changes. We focused on two distinct periods: 1978-1999 and 2000-2021.

## METHODS

We conducted a comprehensive time-series study using secondary national data on malaria-associated morbidity and mortality in Colombia, covering the extensive period from 1978 to 2021. The data used in this study were obtained from secondary sources. Specifically, for the period 1978-2007, the data were sourced from information provided by the direct campaigns of the Ministry of Health and the Malaria Prevention and Control Program. Subsequently, from 2008 to 2021, data were sourced from the Integrated Social Protection Information System (https://www.sispro.gov.co/Pages/Home.aspx). Official projections from the National Administrative Department of Statistics (DANE) and estimates from the Malaria Program based on the 1971, 1985, 1993, and 2005 censuses were used to obtain information on national and regional annual populations. The analysis included spatiotemporal and population variables, focusing on confirmed cases of malaria and associated deaths at both national and regional levels. 

The official surveillance protocol outlined in the national prevention and control program was followed. Malaria diagnosis primarily relies on thick smears as the gold standard. Rapid tests are also part of the diagnostic process. The Ministry of Health and Social Protection provides free treatment for malaria cases^7^. 

A database was created using Microsoft Excel (Microsoft, Redmond, WA, USA) to collect, store, and tabulate the study variables. Absolute frequency measures were included, such as the accumulated load of general cases by region and municipality, and relative frequencies, such as the annual parasite index (API) per 1,000 inhabitants, as along with the annual *Plasmodium falciparum* index (AFI) and annual *Plasmodium vivax* index (AVI). These relative frequencies were calculated by dividing the number of cases by the number of parasite species per 1,000 inhabitants in the at-risk population. 

Malaria transmission risk stratification was conducted nationwide during 1978-1999 and 2000-2021 by adopting and adapting the criteria set forth by the Pan-American Health Organization for risk area stratification. Trends in both API and general mortality rates were analyzed for national and regional malaria between 1978 and 2021. To analyze the trends in the API, AFI, AVI, and age-standardized malaria-associated mortality rates, a Joinpoint regression model was employed^8^. After identifying the trend, the Joinpoint regression model was used to estimate the annual percentage change (APC) with its corresponding 95% confidence interval^9^. Additionally, the Joinpoint regression model was used to calculate the average annual percentage change (AAPC) as a summary measure over a fixed interval. A p-value less than 0.05 was considered significant.

In this study, the response variables AFI and AVI were used to assess morbidity, whereas the age-standardized mortality rate was the response variable for mortality. Year was used as the regressor variable for both the morbidity and mortality analyses, thus representing the progression of time throughout the study period. 

This study strictly adhered to the ethical requirements outlined in Resolution 8430 of 1993 (Article 11) of the Ministry of Health and Social Protection of Colombia. Accordingly, our research was classified as risk-free; therefore, an approval from the ethics committee was unnecessary^10^. Moreover, to protect the privacy and confidentiality of the individuals included, the databases were anonymized.

## RESULTS

### ● Malaria-associated morbidity in Colombia during 1978-2021

**FIGURE 1:**
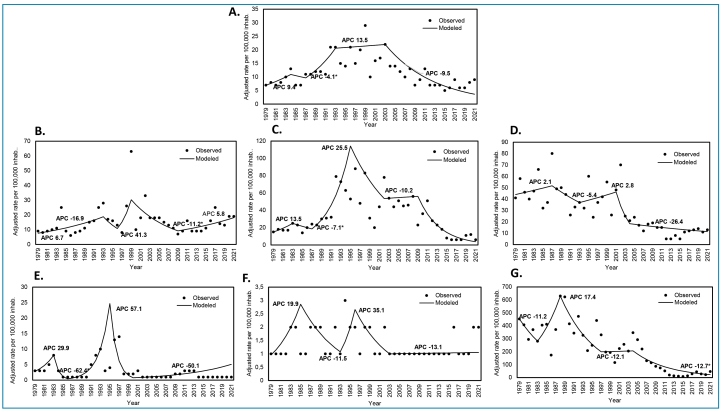
Malaria-associated morbidity trend in Colombia and eco-epidemiological regions, 1978-2021. **A:** Colombia, **B:** Pacific, **C:** Uraba-Bajo Cauca-Sinu-San Jorge, **D:** Amazonia, **E:** Caribbean, **F:** Andean, **G:** Orinoco.

A total of 4,505,552 malaria cases have been registered in the country, with an average of 102,399 cases per year. Fifty-five percent of cases (2,477,623/4,505,552) were registered between 1978 and 1999. In total, 62.4% (2,683,509/4,505,552) of these infections were caused by the species *P. vivax*. On comparing the periods of 1978-1999 and 2000-2021, the absolute number of cases (449,694) of endemic malaria reduced by 18% (Table 1). Analysis of malarial morbidity in Colombia showed a significant contrast between the AAPCs of the two periods. By 1999, AAPC increased to 5.1 (95% CI: 3.7 to 7.1). In contrast, the number of malaria cases decreased significantly during 2000-­2021, with an AAPC of −4.6 (95% CI: −6.3 to −2.8; Table 2 **).** In general, malaria-associated morbidity exhibited a variable endemic-epidemic pattern during the study period, with a marked upward trend between 1978 and 1999, followed by a progressive decline from the first decade of this century until 2021 (Figure 1). 

**TABLE 1:** Dynamics of malaria cases: Comparison periods, strata, and eco-epidemiological regions in Colombia (1978-1999 and 2000-2021).

Region	1978-1999			2000-2021			% Variation
	S4	S3	TOTAL	S4	S3	TOTAL	
**Pacific**	658,830	13,446	672,276	1,067,095	4,660	1,071,755	59%
**Uraba**	1,064,731	32,930	1,097,661	617,223	23,625	640,848	−42%
**Amazonia**	303,953	15,998	319,950	149,268	6,137	155,405	−51%
**Orinoco**	161,702	42,984	204,686	27,918	7,199	35,117	−83%
**Caribbean**	98,385	24,596	122,981	62,818	14,970	77,788	−37%
**Andean**	45,652	14,416	60,069	35,839	11,178	47,017	−22%
**TOTAL**	2,333,253	144,370	2,477,623	1,960,160	67,769	2,027,929	−18%

**Uraba:** Uraba-Bajo Cauca-Sinu-San Jorge; S3: Stratum 3 (non-active and residual foci); S4: Stratum 4 (active foci); **bold:** percentage variation of the total cases.

**TABLE 2:** Annual parasite index and standardized mortality rate, with the average annual percent change in malaria indicators in Colombia, 1978-1999 and 2000-2021.

	API (per 1,000)				Standardized death rate (per 100,000)			
Eco-epidemiological aspects	1978-1999	AAPC (95% CI)	2000-2021	AAPC (95% CI)	1978-1999	AAPC (95% CI)	2000-2021	AAPC (95% CI)
National	11.5	5.1*	9	−4.6*	0.9	−10.3*	0.1	−9.4*
	(8-15)	**(3.7 to 7.1)**	(7-13)	**(−6.3 to −2.8)**	(0.8-1.0)	(−12.1 to −8.4)	(0.05-0.2)	**(−15.7 to −2.5)**
Pacific	11.2	4.5	15.5	−0.5	1.32	−4.6	0.40	−9.8*
	(9-17)	(0.9 to 8.2)	(11-18)	(−3.2 to 2.2)	(1.21-1.44)	(−9.8 to 1.0)	(0.33-0.43)	**(−17.2 to −1.7)**
Uraba	27.5	6.6*	25.5	−12.5*	0.76	−14.1*	0.17	−12.9*
	(20-53)	**(3.4 to 10)**	(8-46)	**(−15.1 to −9.8)**	(0.68-0.82)	**(−17.4 to −10.8)**	(0.10-0.27)	(−15.3 to −10.4)
Amazonia	41.5	−1.7*	13.5	−6.2*	2.73	-	0.42	-
	(33-50)	**(−3.7 to −1.4)**	(11-19)	**(−9.5 to −2.8)**	(2.31-3.18)		(0.34-0.53)	
Orinoco	356.5	−3.0*	49.5	−11.2*	2.13	-	0.23	-
	(249-415)	**(−5.5 to −0.3)**	(23-205)	**(−17.2 to −4.8)**	(1.83-2.44)		(0.15-0.32)	
Caribbean	3.4	2.5	2.2	−1.6	0.12	-	0.04	-
	(1.5-6.4)	(−3.7 to 9.0)	(1.3-3.5)	(−7.1 to 4.3)	(0.05-0.19)		(0.00-0.09)	
Andean	1.7	1.8	0.9	1.0	0.30	-	0.08	-
	(1.4-2.8)	(−0.8 to 4.5)	(0.6-1.3)	(−0.9 to 3.0)	(0.23-0.37)		(0.00-0.14)	

* p < 0.05. **Uraba:** Uraba-Bajo Cauca-Sinu-San Jorge; **AAPC:** average annual percentage change; **API:** annual parasite index; **95% CI:** 95% confidence interval.

The APC during 1978-1983 was 9.4, when morbidity exhibited an upward trend. This upward trend continued between 1986 and 1992, with an APC of 13.5; subsequently, the morbidity decreased between 2002 and 2021, and the APC was −9.5 (Figure 1). The Orinoco and Amazonia regions presented a marked reduction of morbidity, particularly during the second period. The AAPCs for these regions were −12.7 and −26.4, respectively. However, during the first period, the Uraba region had the highest APC (25.5). Subsequently, a marked and sustained decline was observed until the end of the study period. In contrast, the Pacific region registered a gradual decrease until 2012 (APC −11.2) and a subsequent increase that is maintained to date (APC 5.8) (Figure 1).

*P. vivax* infections predominated the malarial infections in most years (86.4% [38/44]) during the entire study period, except for 1998, when *P. falciparum* infections predominated. As of 2014, the proportion of *P. falciparum*-associated infections equalized to that of *P. vivax*-associated infections.

Malaria was considered endemic during 33 years and epidemic during 11 years of the 44 years of study. The cumulative burden of malaria cases during the years of endemic malaria was 2,849,850, equivalent to an annual average of 86,359 cases. *P. vivax* was the most prevalent malarial infectious agent during these years, at 63% (1,802,403/2,849,850). The median value of the annual parasite index (mAPI) in years during which malaria was an endemic was 10.7 per 1,000 inhabitants, with maximum and minimum values of 20.4 and 6.7 per 1,000 inhabitants, respectively. The accumulated burden of epidemic cases registered during the period 1978-2021 was 1,626,919, corresponding to an annual average of 197,902 cases for years during which malaria was an epidemic. The highest accumulated burden of endemic cases occurred during the first period (916,108 cases), and the frequency of epidemic outbreaks was 4.5 years. *P. vivax* infections predominated (62.5%, 611,427 of 1,629,919 cases) during the years in which malaria was epidemic. The mAPI in the years of epidemic malaria was 14.2 per 1,000 inhabitants, with maximum and minimum values of 29.3 and 8.5 per 1,000 inhabitants, respectively. Between 2010 and 2021, the most vulnerable population groups were economically active individuals between 15 and 29 years of age (35.8%, 290,499), followed by those under 15 years of age (31.9%, 258,717).

### ● Behavior by eco-epidemiological region

According to the distribution of the accumulated burden by eco-epidemiological region, 78.9% (1,876,821 cases) was concentrated in the regions of Uraba-Bajo Cauca-Sinu-San Jorge (38, 8%), the Pacific (23.8%), and Amazonia (16.3%) (Table 1).

**FIGURE 2:**
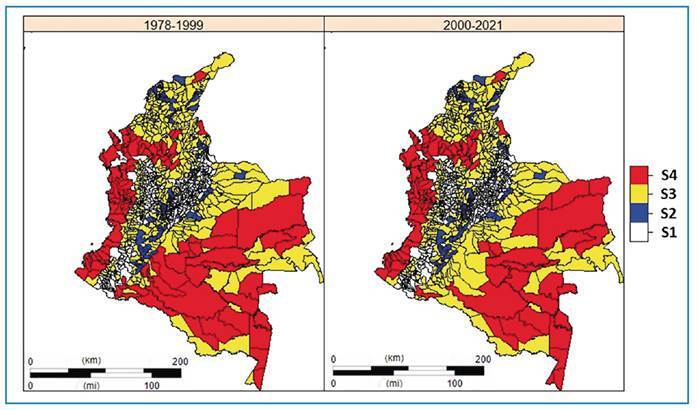
Distribution of the risk of malaria transmission in Colombia, 1978-1999 and 2000-2021. **S1:** Stratum 1 non-receptive; **S2:** Stratum 2 receptive; **S3:** Stratum 3 (non-active and residual foci); **S4:** Stratum 4 (active foci).

At the end of the 20th century, the number of municipalities with active autochthonous S4 transmission was lower (n = 97); however, they registered a higher accumulated case burden. From 2000-2021, the number of S4 municipalities was higher (n = 107) than in the previous period. Relevant regional changes were observed between the first and second periods. During the initial period, a regional focus with active transmission was noted, located between the border municipalities of the Amazonia (Caqueta, Putumayo, and Amazonas) and Orinoco (Meta) regions; between 2000 and 2021, transmission changed from the S4 to S3 regions (Figure 2).

**FIGURE 3:**
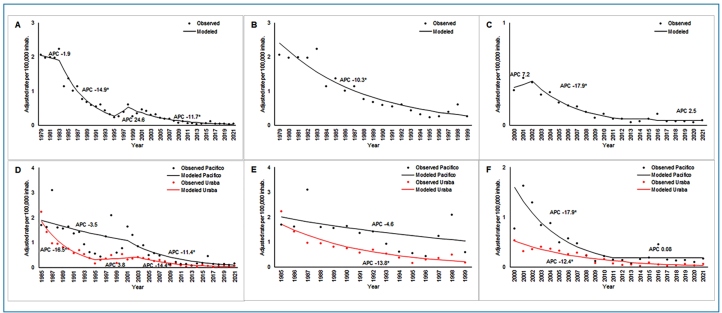
Malaria-associated mortality trends in Colombia during 1978-2021 and in the Uraba-Bajo Cauca-Sinu-San Jorge and Pacific regions during 1985-2021. **A:** Colombia 1978-2021; **B**: Colombia 1978-1999; **C:** Colombia 2000-2021; **D:** Uraba-Bajo Cauca-Sinu-San Jorge and Pacific regions 1985-2021; **E:** Uraba-Bajo Cauca-Sinu-San Jorge and Pacific regions 1985-1999; **F:** Uraba-Bajo Cauca-Sinu-San Jorge and Pacific regions 2000-2021.

Within the Uraba-Bajo Cauca-Sinu-San Jorge eco-epidemiological region, there was a notable difference in the distribution of cases between the first and second periods. In the first period, there was a high concentration of cases in the S4 municipalities compared to other municipalities in the Uraba subregion. However, during the second period, there was a significant spread of transmission observed in other S4 municipalities in the Bajo Cauca subregion. Similar changes were observed in the Andean region, where during the first period, spatial concentration of cases was high in the Pueblo Rico focus (Risaralda); during 2000-2021, this was more evident in the Tibu region (Norte de Santander) (Figure 3).

### ● Malaria-associated mortality in Colombia during 1978-2021

A total of 7,608 deaths due to malaria were recorded, corresponding to 177 annual deaths (7,608/43 years), and the median number of deaths was 123. In absolute numbers, malaria-related deaths showed a downward trend. A total of 78% (6242/7608) of all malarial deaths occurred during 1978-1999, and the number of malaria-associated deaths decreased from 529 in 1979 to 25 in 2021. The analysis of malaria-associated mortality in Colombia indicated a significant decreasing trend in both periods. Between 1978 and 1999, there was a noteworthy decline with an AAPC of −10.3 (95% CI: −12.1 to −8.4). Similarly, the number of malaria cases reduced substantially from 2000 to 2021, with an AAPC of −9.4 (95% CI: −15.7 to −2.5; Table 2 **)**.

Deaths occurred mainly among those aged < 24 years (57%). Deaths of children under 5 years of age accounted for 26.4% and deaths of those between 5 and 14 years of age accounted for 14.0% of the total malaria-related deaths in Colombia. The crude malaria-associated mortality rate for the study period was 0.451 deaths per 100,000 inhabitants, and the age-adjusted rate was 0.446. Between 1978 and 1983, the number of deaths exhibited a downward trend, with an APC of 1.9; from 1983 to 1995, there was an accelerated and statistically significant downward trend (APC: −14.9); between 1995 and 1998, the trend was upward, with an APC of 24.6; and between 1998 and 2021, the trend reversed again, with an APC of −11.7, which was statistically significant (Figure 3).

A total of 1,939 deaths from malaria were reported in the Pacific region between 1985 and 2021. At the national level, deaths among those under 24 years of age predominated and accounted for 54.5% of all deaths in the region. Deaths of children under five years of age accounted for 21.7% and deaths of those between 5 and 14 years of age accounted for 16.5% of the total deaths from malaria. Between 1985 and 2001, mortality presented a downward trend with an APC of −3.5, and from 2001 to 2021, mortality continued its downward trend with an APC of −11.4, which was statistically significant (Figure 3).

Between 1985 and 2021, 959 malaria-related deaths were registered in the Uraba-Bajo Cauca-Sinu-San Jorge region. In this region, deaths predominantly occurred among those under 24 years of age, accounting for 56.3% of all deaths. Deaths of children under five years of age and those between 5 and 14 years of age accounted for 23.0% and 14.1% of the total deaths from malaria, respectively. The deaths in the Uraba region showed an accelerated downward trend between 1985 and 1995, with an APC of −16.5, which was statistically significant (Figure 3).

## DISCUSSION

This study revealed some changes regarding the behavior of malaria in Colombia during the study period. Although new cases reduced slightly at the expense of the endemic component, the frequency and intensity of epidemic transmission were maintained. In addition, infections caused by *P. vivax* maintained their historical predominance in most years. The distribution and regional extension of risk also started increasing in the active transmission strata of the national territory at the beginning of the second period. Finally, malaria-associated mortality declined in a drastically and sustained manner between 1978 and 2021.

A spectrum of extreme malaria transmission situations is currently observed in the Americas. In 2021, Brazil, Colombia, and the Bolivarian Republic of Venezuela contributed to 79% of the regional cases, and countries such as El Salvador, Argentina, and Paraguay were certified by the World Health Organization as malaria-free in 2018, 2019, and 2021, respectively^1^.

This scenario is common in malaria-endemic countries with low and unstable transmission in Asia, the Middle East, some African countries, and the Americas^11^. Endemics of all possible degrees are recorded in these regions, which are markedly influenced by seasonal changes; the yearly incidences of malaria and the characteristics of the endemic-epidemic fluctuated markedly. The population immunity was variable, and competent vectors with low biting rates and different feeding habits (zoophilic and/or anthropophilic) dominated^12^. 

The reduction in malaria endemicity observed in the country, mainly in recent decades, is likely to have contributed to the increase in mosquito net coverage, consolidation of surveillance actions, expansion of diagnosis, and timely treatment of the main foci of active transmission with a higher case-load. This situation coincides with better optimization of resources by the national program and the opportunity to improve local capacity by implementing prevention and control projects in priority areas, financed with resources from the Global Fund^13^.

In turn, the persistence of the frequency and intensity of malaria epidemic cycles in the national territory has been determined by the maintenance and recurrence of complex interactions and economic, social, political, general, and cultural contradictions that occur in regional endemic scenarios^14^ ^,^ ^15^. The triggers that contribute to the perpetuation of malaria epidemic cycles are the illegal, intensive, and irrational exploitation of natural and mineral resources in endemic receptive regions, such as gold mining in the jungle regions^16^.

Similar situations are observed in endemic countries in the Americas with the greatest disease burden, such as Brazil, Venezuela, and Peru. For example, the risk of disease prevalence in miners who worked or lived in areas of gold extraction in camps north of Mato Grosso in the Brazilian Amazon was different from others in Barbieri and Oya-Sawyer^17^. Similarly, in Madre de Dios in southern Peruvian Amazonia, there was a resurgence of malaria, accompanied by ecological, political, and socioeconomic changes related to the proliferation of illegal gold mining. Furthermore, the malaria transmission pattern was unstable, with a heterogeneous spatial distribution and strong association with illegal gold mining^18^. Likewise, the Venezuelan Society of Public Health describes the uncontrolled increase in illegal mining in southern Bolivar State as a major determinant of the re-emergence of malaria in Venezuela^19^. In Colombia, Castellanos et al. established an association between mining and malaria in endemic areas, with active transmission in Colombian departments having the greatest malaria burdens^20^.

Another determinant that has contributed to the dynamics of malaria transmission in the country was the expansion and concentration of illicit crop areas in endemic, isolated regions with abundant plant biomass and great water wealth. In addition, this activity attracts susceptible populations in search of job opportunities, whose interactions between carriers and infected mosquitoes trigger frequent seasonal outbreaks. Additionally, they cause population dispersion to flow toward receptive strata distributed in different eco-epidemiological regions of the national territory^21^. Gómez-López et al. managed to establish an ecological association in Guaviare between the periods of crisis of coca bonanzas and the increased incidence of malaria cases^22^. In addition, contextual climatic factors, such as the El Niño phenomenon, were strongly associated with the appearance of malaria epidemic outbreaks. The largest historical malaria epidemic recorded in the country occurred in 1998, and this association has also been documented in other years of epidemic malarial occurence^23^.

The changes observed in the parasite formula at the end of the study period, with a predominance of *P. falciparum* infections, could be explained by the intensification and concentration of epidemic transmission. In recent years, this has been observed mainly in the Pacific region, an area with a high prevalence of *P. falciparum* and a predominance of Afro-descendant populations^24^. In addition, settler populations migrated from various regions of the country to the Bajo Cauca Antioqueño subregion and re-emerged foci in the Colombian Amazonia^25^. Marked deforestation, deterioration of jungle areas, and alteration of their main hydrographic basins caused by illegal economic activities conducted by different armed and illegal actors have been observed in these eco-epidemiological regions^26^. Notably, the young and economically active population is the most vulnerable demographic group, mainly during epidemics^27^ ^,^ ^28^.

In the eco-epidemiological regions of malaria transmission in the national territory, a variety of ecotypes are distributed that favor the presence of *Anopheles* vectors, such as jungles, alluvial plains, coasts, savannahs, gallery forests, tropical dry forests, foothills, mountains, mangroves, and foothills. The main malaria vectors in Colombia are *Anopheles albimanus*, *An. darlingi*, and *An. nuneztovari*. These mosquito species are responsible for the disease transmission throughout the country. *An. albimanus* is widely distributed across Colombia, adaptable to both coastal and inland regions, and prevalent in tropical and subtropical areas. *An. darlingi*, which is found in the Amazon rainforest, is one of the most efficient malaria vectors in Latin America, breeding in slow-moving rivers and streams. *An. nuneztovari* is primarily found in the Amazonas, Vaupes, and Guainia departments and thrives in humid areas with dense vegetation^29^ ^,^ ^30^.

A sustained secular reduction in malarial mortality was notable in both periods, especially in the most recent one. The gradual strengthening of the prevention and control program has likely contributed to this through projects with external financing. The funding in turn provided the opportunity to improve and expand the diagnostic network with microscopy and complement it with the use of rapid diagnostic tests in hard-to-reach areas and timely and effective treatment with artemisinin derivatives^31^ ^,^ ^32^. However, in the eco-epidemiological regions with the highest frequency of malarial deaths, the deaths generally occur in years where epidemic situations were observed in dispersed and difficult-to-access rural areas with weaknesses in the health care network^33^ ^,^ ^34^.

Over the years, Colombia has witnessed an evolution in antimalarial regimens as a part of its efforts to combat malaria. Chloroquine was initially used as the primary treatment for this disease. However, owing to the emergence of chloroquine-resistant strains of *P. falciparum*, the country had to adopt this approach. The national malaria program then shifted to artemisinin-based combination therapies as the first-line treatment. Artemisinin is highly effective in curing uncomplicated malaria and reducing the risk of developing drug resistance^35^. However, despite these advancements, Colombia faces challenges in drug resistance, particularly to the antimalarial drug sulfadoxine-pyrimethamine^35^. 

Some limitations in this study are related to the quality and reliability of the information available during the study period. They are primarily related to the uniformity and completeness of fundamental variables of morbidity and mortality, which were available in the databases of the information systems utilized during the study period. However, the selected secondary sources were considered reputable and authoritative for capturing the required information. The creation of new municipalities segregated from endemic municipalities partly explains the greater extension of the foci of active transmission. Although official death certificates from DANE were used as the source of mortality, the definition of death from malaria was too sensitive and flexible and might have included unconfirmed cases as deaths from malaria, limiting objective comparisons at different times. The lack of specific information on deaths by parasitic species in the DANE data limits our ability to provide a detailed breakdown of mortality statistics for malaria. However, we believe that the findings of this study constitute the best available technical evidence that should be considered by decision makers and those responsible for the national malaria prevention and control program. 

Although a reduction in endemic levels was observed during the study period in the malaria-endemic areas of the national territory, the frequency and intensity of epidemic transmission were maintained. The dynamics and extent of the risk in priority eco-epidemiological regions with active transmission have changed. Currently, there is an equalization or predominance of the proportion of infections caused by *P. falciparum*, and the downward secular trend brought about by malaria-related mortality continues throughout the country.
